# Case Report: Tislelizumab-associated secondary adrenal insufficiency in a patient with lung squamous cell carcinoma

**DOI:** 10.3389/fendo.2026.1915283

**Published:** 2026-09-18

**Authors:** Zongsheng Tian, Guanghui Zhang, Wenli Kong, Xueliang Xu

**Affiliations:** 1 Department of Respiratory and Critical Care Medicine, Linyi People’s Hospital, Linyi, China; 2 Department of Hyperbaric Oxygen Medicine, Linyi People’s Hospital, Linyi, China

**Keywords:** adrenal crisis, immune checkpoint inhibitors, immune-related adverse events, lung squamous cell carcinoma, secondary adrenal insufficiency, tislelizumab

## Abstract

**Background:**

Immune checkpoint inhibitors (ICIs) have transformed cancer treatment, but immune-related adverse events remain clinically challenging. Tislelizumab is a humanized anti-PD-1 monoclonal antibody widely used in China, and tislelizumab-associated secondary adrenal insufficiency (SAI) remains insufficiently characterized, particularly with regard to how and when it is recognized.

**Case presentation:**

A 66-year-old man underwent video-assisted thoracoscopic surgery for right lower lobe squamous cell carcinoma (pT1cN1M0, stage IIB), followed by four cycles of carboplatin, paclitaxel liposome and tislelizumab, then 13 cycles of tislelizumab maintenance. His last tislelizumab dose was administered on December 11, 2023. Poor appetite and fatigue first developed in November 2023, accompanied by a blood pressure of 92/57 mmHg. His symptoms improved after supportive treatment, but adrenocorticotropic hormone (ACTH) and cortisol were not measured. He subsequently developed headache, nausea, vomiting, and progressive weight loss of 19 kg. In November 2024, he was admitted with hypotension (75/56 mmHg) and severe hyponatremia (108.9 mmol/L); chest CT and bronchoscopy excluded tumor recurrence, and he improved with supportive care. Symptoms recurred within a week of discharge, and he was readmitted in December 2024 with hypotension (52/37 mmHg) and recurrent hyponatremia. On December 11, 2024, approximately 13 months after the initial symptoms, morning cortisol was <1.00 µg/dL and ACTH was inappropriately low at <5.00 pg/mL, supporting SAI (Naranjo score 6, probable). Pituitary MRI, cosyntropin stimulation testing and comprehensive pituitary hormonal assessment were unavailable; hypophysitis was therefore not confirmed, and the site of hypothalamic-pituitary-adrenal (HPA) axis injury could not be determined. He improved with intravenous hydrocortisone and fluids and was transitioned to oral prednisone. ACTH and cortisol remained low through July 2025 during replacement, although endogenous HPA-axis recovery could not be reliably assessed. At telephone follow-up in June 2026 no recurrent adrenal crisis was reported.

**Conclusions:**

Tislelizumab-associated SAI may present with nonspecific symptoms and remain unrecognized until adrenal crisis develops, including months after the last dose. Unexplained fatigue, gastrointestinal symptoms, weight loss, hyponatremia, hypoglycemia, or hypotension should prompt early measurement of paired morning ACTH and cortisol levels. Prompt glucocorticoid replacement and structured endocrine follow-up are essential for patient safety and assessment of potential HPA-axis recovery.

## Introduction

Immune checkpoint inhibitors (ICIs) targeting PD-1/PD-L1 and CTLA-4 have transformed oncology practice (1, 2), yet their use is associated with immune-related adverse events (irAEs) affecting multiple organ systems (3). Endocrine irAEs are clinically important because they can lead to persistent, and sometimes permanent, hormone deficiencies requiring long-term or lifelong hormone replacement, even after discontinuation of ICI therapy (4). Among these, secondary adrenal insufficiency (SAI) associated with ICI-related pituitary-adrenal axis dysfunction is uncommon but potentially life-threatening if not promptly recognized (5).

Tislelizumab is a humanized IgG4 anti-PD-1 monoclonal antibody developed in China and specifically engineered to minimize Fcγ receptor binding on macrophages, thereby reducing Fc-mediated effects that may compromise antitumor T-cell activity. It received its first regulatory approval in China in December 2019 for relapsed or refractory classical Hodgkin lymphoma and has since been approved for multiple hematologic and solid tumor indications (6). Although safety data on tislelizumab have accumulated rapidly, reports specifically addressing tislelizumab-associated adrenal insufficiency remain relatively few and are heterogeneously reported.

Herein, we report a case of SAI following tislelizumab treatment for resected lung squamous cell carcinoma. The patient was diagnosed with SAI during an episode suggestive of adrenal crisis after prior exposure to tislelizumab, and follow-up ACTH and cortisol measurements obtained during glucocorticoid replacement remained low, although endogenous HPA-axis recovery could not be formally assessed. This case highlights the need to consider tislelizumab-associated SAI in patients with current or prior exposure to ICIs and supports structured endocrine surveillance during and after ICI therapy.

## Case description

### Patient history and clinical course

A 66-year-old male farmer was diagnosed with right lower lobe squamous cell carcinoma in May 2022. He underwent video-assisted thoracoscopic surgery (VATS) with right middle and lower lobectomy plus lymph node dissection (postoperative stage: pT1cN1M0, stage IIB). Adjuvant therapy comprised four cycles of carboplatin(0.5 g), paclitaxel liposome(210 mg), and tislelizumab (200 mg), followed by 13 cycles of tislelizumab monotherapy maintenance (200 mg every 4 weeks), with the last dose administered on December 11, 2023.PD-L1 expression was not tested before treatment. His medical history was otherwise unremarkable, except for a 40-pack-year smoking history. No history of autoimmune disease was documented. Apart from single-dose dexamethasone 5 mg administered intravenously as premedication with paclitaxel liposome on the day of each of the four combination chemotherapy cycles, he had received no systemic glucocorticoid therapy before the diagnosis of adrenal insufficiency; no glucocorticoid premedication was given during the subsequent 13 cycles of tislelizumab monotherapy maintenance. No systematic irAE-oriented endocrine baseline assessment was performed before tislelizumab initiation; pretreatment ACTH, cortisol, and gonadal hormones were unavailable. Preoperative echocardiography showed no clinically significant abnormalities. Thyroid function was assessed at four time points during and after treatment (**Table 1**). Isolated borderline abnormalities were observed while on therapy: a marginally low thyroid-stimulating hormone (TSH) of 0.524 µIU/mL (reference range, 0.55–4.78) in December 2022, a marginally low free thyroxine (FT4) of 11.00 pmol/L (reference range, 11.5–22.7) in August 2023, and a mildly elevated free triiodothyronine (FT3) of 7.15 pmol/L (reference range, 3.5–6.5) in December 2023. On each occasion the remaining thyroid parameters were within the reference range, and none of these abnormalities was accompanied by clinical thyroid dysfunction or required treatment. Thyroid peroxidase antibodies (TPO-Ab) were elevated in December 2022 (300.4 IU/mL; reference range, 0–60) and August 2023 (256.2 IU/mL), and declined to 119.6 IU/mL in December 2023; TPO-Ab was not repeated thereafter. At the first admission in November 2024, FT3, FT4 and TSH were all within the reference range. These findings indicate thyroid autoantibody positivity with minor, transient thyroid-function abnormalities but no overt thyroid dysfunction. Because pretreatment TPO-Ab testing was unavailable, whether the thyroid autoimmunity predated tislelizumab could not be determined.

**Table 1:** Key laboratory findings during hospitalization and follow-up.

Parameter	Dec 11, 2022	Aug 10, 2023	Dec 24, 2023	1st admission (Nov 16, 2024)	Readmission/diagnosis (Dec 11, 2024)	Follow-up (Feb 11, 2025)	Follow-up (Jul 8, 2025)	Reference range
ACTH (8:00 AM)	NM	NM	NM	NM	<5.00	<5.00	<5.00 pg/mL	0–46 pg/mL
Cortisol (8:00 AM)	NM	NM	NM	NM	<1.00	1.35	<1.00	5-25 µg/dL
Sodium	NM	Normal	135	108.9	122.6	Normal	Normal	137–147 mmol/L
Potassium	NM	Normal	Normal	Normal	Normal	Normal	Normal	3.5-5.3 mmol/L
FT3	5.11	5.05	7.15	5.33	NM	NM	NM	3.5-6.5 pmol/L
FT4	11.81	11.00	12.78	15.16	NM	NM	NM	11.5-22.7 pmol/L
TSH	0.524	0.942	1.687	4.321	NM	NM	NM	0.55-4.78 uIU/mL
TPO-Ab	300.40	256.20	119.60	NM	NM	NM	NM	0–60 IU/mL

ACTH, adrenocorticotropic hormone; FT3, free triiodothyronine; FT4, free thyroxine; NM, not measured; TSH, thyroid-stimulating hormone; TPO-Ab, thyroid peroxidase antibody. Reference ranges are shown according to the reporting laboratory. Follow-up ACTH and cortisol measurements were obtained during glucocorticoid replacement, and sampling relative to prednisone administration was not standardized.

### Clinical presentation and diagnosis

The patient first developed poor appetite and fatigue in November 2023. At that time, his temperature was 36.3 °C, pulse rate 86 beats/min, respiratory rate 21 breaths/min, and blood pressure 92/57 mmHg. His symptoms improved following symptomatic and supportive treatment, but cortisol and ACTH levels were not measured.

On November 16, 2024, the patient was admitted with progressive fatigue, anorexia, nausea and vomiting. His temperature was 36.2 °C, pulse rate 76 beats/min, respiratory rate 20 breaths/min, and blood pressure 75/56 mmHg. He was alert and cooperative, with unlabored breathing, coherent responses, and clear speech. Breath sounds were slightly coarse without wheezes or crackles. Cardiac rhythm was regular without pathological murmurs. The abdomen was soft and non-tender without rebound tenderness, and no lower-extremity edema was observed. Laboratory findings revealed severe hyponatremia (108.9 mmol/L), hypochloremia (78.3 mmol/L), and hypoglycemia (3.38 mmol/L). Given his history of lung cancer and prior immunotherapy, disease progression and treatment-related toxicity were initially considered. Chest CT and bronchoscopic evaluation showed postoperative changes without evidence of tumor recurrence or metastasis. He was treated with antibiotics, fluid resuscitation, and sodium supplementation, with symptomatic improvement, and was discharged on November 24, 2024.

However, his symptoms recurred and worsened within one week after discharge, leading to readmission on December 4, 2024. On readmission, his temperature was 36.2 °C, pulse rate 85 beats/min, respiratory rate 16 breaths/min, and blood pressure 52/37 mmHg, raising concern for adrenal crisis. He was alert but appeared fatigued, and the remainder of the physical examination was essentially unchanged from that documented during the first admission. Repeat laboratory testing showed recurrent hyponatremia, with serum sodium declining from 136.9 mmol/L on readmission to 122.6 mmol/L by December 11. In view of recurrent hypotension, hyponatremia, profound weight loss, and the absence of tumor recurrence, endocrinology consultation was obtained. Morning endocrine testing on December 11 showed cortisol <1.00 µg/dL (reference range, 5-25 µg/dL) and an inappropriately low ACTH level of <5.00 pg/mL (reference range, 0–46 pg/mL), supporting the diagnosis of secondary adrenal insufficiency. Thyroid function, last measured at the first admission on November 16, 2024, had been normal (**Table 1**). Non-contrast head CT showed no brain metastasis or pituitary macroadenoma. Pituitary MRI was not performed during the acute illness because of the patient’s poor general condition. Key laboratory findings across both admissions and follow-up are presented in **Table 1**. The overall clinical course is summarized in **Figure 1**.

**Figure 1:**
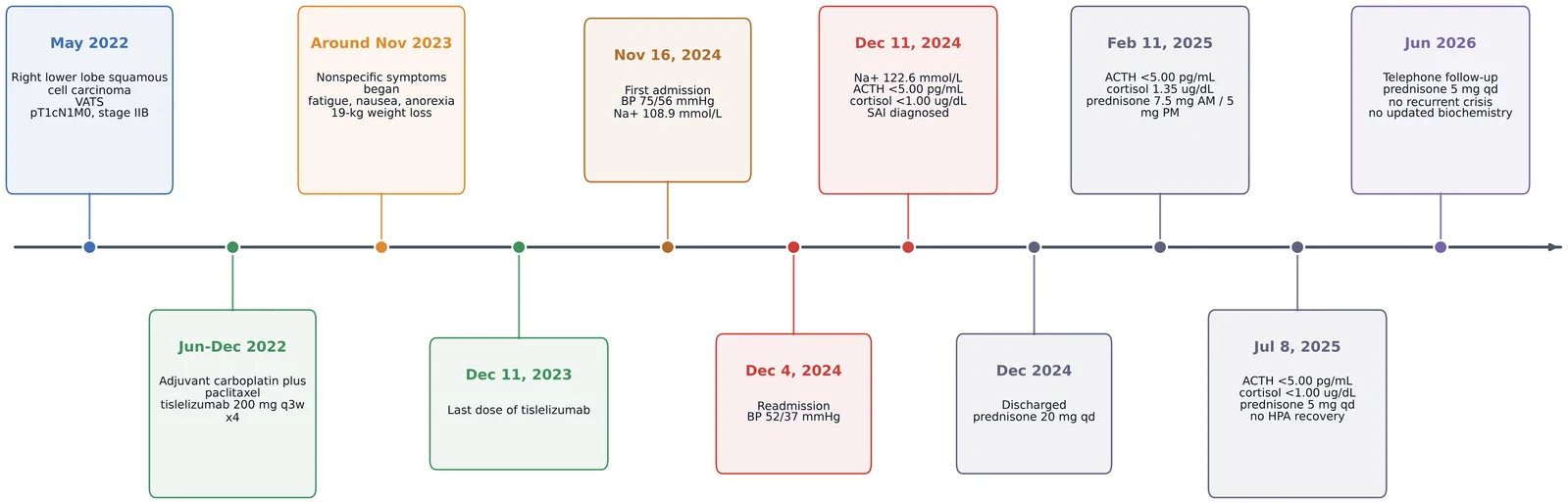
Clinical timeline of tislelizumab-associated secondary adrenal insufficiency. The figure shows the approximately 13-month interval between the first symptoms attributable to adrenal insufficiency and biochemical diagnosis. The colored entries mark the two occasions on which hypotension, and on the second occasion severe hyponatremia, were documented without measurement of paired morning ACTH and cortisol. ACTH, adrenocorticotropic hormone; BP, blood pressure; HPA, hypothalamic-pituitary-adrenal; SAI, secondary adrenal insufficiency; VATS, video-assisted thoracoscopic surgery.

### Diagnostic assessment

The profoundly low morning cortisol level accompanied by an inappropriately low ACTH level strongly supported secondary adrenal insufficiency. Primary adrenal insufficiency was considered unlikely because ACTH was low rather than elevated; in primary adrenal insufficiency, low cortisol is typically accompanied by elevated ACTH (7). Thyroid function measured at the first admission in November 2024 was normal and there was no clinical evidence of thyroid dysfunction at diagnosis, making concomitant thyroid axis involvement unlikely; thyroid hormones were not repeated on December 11, 2024. Chest CT and bronchoscopic evaluation showed no evidence of tumor recurrence or metastasis, and there was no clinical evidence of adrenal or pituitary metastasis. The patient’s only systemic glucocorticoid exposure was single-dose dexamethasone 5 mg given intravenously as chemotherapy premedication on the day of each of the four combination cycles, which had been completed more than two years before diagnosis; such brief, intermittent, low-dose exposure is not expected to produce sustained suppression of the hypothalamic-pituitary-adrenal axis, and the entire year of tislelizumab monotherapy maintenance that followed was glucocorticoid-free. Exogenous glucocorticoid-induced adrenal suppression was therefore considered unlikely. Together with the temporal association with tislelizumab exposure and symptom development during the treatment course, these findings supported tislelizumab-associated secondary adrenal insufficiency as the most likely diagnosis. However, because pituitary MRI, cosyntropin testing, and comprehensive pituitary hormonal assessment were unavailable, hypophysitis could not be confirmed, isolated ACTH deficiency could not be distinguished from broader pituitary dysfunction, and the exact site of HPA-axis injury remained uncertain.

Other treatment-related causes were also reviewed. Carboplatin and paclitaxel were considered less likely direct contributors because adrenal insufficiency is not recognized as a typical adverse reaction in their prescribing information. Causality was assessed using the Naranjo Adverse Drug Reaction Probability Scale, with a score of 6 indicating a “probable” adverse drug reaction (**Table 2**) (8).

**Table 2:** Naranjo adverse drug reaction probability scale.

Question	Score
Previous conclusive reports	+1
Reaction appeared when drug administered	+2
Reaction improved when drug discontinued	0
Reaction reappeared when drug readministered	0
Alternative causes excluded	+2
Placebo rechallenge	0
Drug detected at toxic concentrations	0
Reaction more severe with dose increase	0
Similar reaction to same/similar drug	0
Objective evidence confirmed	+1
Total	6 (Probable)

### Therapeutic intervention, follow-up, and outcomes

After SAI was diagnosed on December 11, 2024, the patient received fluid resuscitation and intravenous hydrocortisone (50 mg in the morning and 25 mg in the evening), followed by transition to oral prednisone replacement. His blood pressure, serum sodium, and clinical symptoms improved, and he was discharged on oral glucocorticoid replacement. During follow-up, prednisone was adjusted to a maintenance regimen according to clinical status. ACTH and cortisol remained low through July 2025 during glucocorticoid replacement, with normalized electrolytes, however, HPA-axis recovery could not be reliably assessed because sampling was not standardized relative to prednisone dosing and dynamic testing was unavailable. At telephone follow-up in June 2026, no recurrent adrenal crisis was reported, but updated biochemical reassessment was not available.

## Discussion

SAI is an uncommon but clinically important endocrine immune-related adverse event associated with ICIs. In this case, the diagnosis was supported by a profoundly low morning cortisol level with an inappropriately low ACTH level, improvement after glucocorticoid replacement, prior exposure to tislelizumab, and the exclusion of tumor recurrence by imaging and bronchoscopic evaluation. Although adrenal insufficiency is a recognized endocrine complication of ICIs, tislelizumab-associated SAI remains insufficiently characterized, particularly with respect to delayed recognition and longitudinal endocrine assessment (5, 9–11).

The mechanism of ICI-associated SAI is thought to involve immune-mediated injury of the pituitary-adrenal axis, presenting as hypophysitis or isolated ACTH deficiency (9, 11), but the two checkpoint pathways appear to differ. For CTLA-4 blockade therapy, pituitary endocrine cells ectopically express CTLA-4, and antibody binding subsequently induces complement-mediated tissue injury (12). No comparable mechanism has been established for PD-1 blockade, although the clinical phenotype differs from that of CTLA-4 blockade. In a comparative series, hypophysitis was less frequent after nivolumab or pembrolizumab than after ipilimumab, pituitary enlargement was uncommon, and the interval to diagnosis was longer (13). Isolated ACTH deficiency can occur after anti-PD-1 therapy even without pituitary enlargement. The low ACTH and cortisol levels in our patient were compatible with this phenotype, although pituitary MRI was unavailable. In a Japanese cohort, pretreatment anti-pituitary antibodies and several HLA alleles (HLA-Cw12, HLA-DR15, HLA-DQ7, and HLA-DPw9) were more frequent in patients who developed ICI-induced isolated ACTH deficiency, suggesting a possible role for host susceptibility (14). Anti-GNAL and anti-ITM2B autoantibodies have also been associated with ICI-related hypophysitis (15). A complementary paraneoplastic mechanism has been proposed in a patient with large-cell neuroendocrine carcinoma, in whom ectopic ACTH expression and lymphocytic infiltration were demonstrated in tumor tissue, together with circulating anti-POMC antibodies recognizing the ACTH25–39 region (16). Tislelizumab is engineered to minimize Fcγ receptor binding on macrophages, but whether this modification affects the risk or pathogenesis of endocrine irAEs remains unknown. In our patient delayed onset and delayed recognition cannot be distinguished, because adrenal function was never assessed during the symptomatic interval. These findings remain preliminary and do not yet support routine antibody or HLA testing, so recognition still depends on paired morning ACTH and cortisol measurement. Future studies should evaluate potential predictive biomarkers to facilitate early detection, prompt treatment, and close monitoring of this potentially life-threatening endocrine irAE.

**Table 3** summarizes 12 patients reported in nine previous publications (17–25). Clinical presentation varied across the reported cases, from biochemical abnormalities found during routine screening to adrenal crisis. Blood pressure was not reported in 8 of the 12 patients, making it difficult to compare the severity of hemodynamic involvement. Cortisol deficiency lasting more than 7–8 months was reported in two patients. Apparent biochemical recovery was reported in only one patient, whose cortisol level decreased again after one cycle of tislelizumab rechallenge. Follow-up in most other patients lasted only about 5 weeks to 3 months. In our patient, anorexia, fatigue, and hypotension (92/57 mmHg) first appeared during tislelizumab treatment in November 2023. The symptoms improved after supportive treatment but later recurred, and SAI was diagnosed approximately 13 months after their initial appearance. During the two admissions, the patient developed profound hyponatremia (108.9 mmol/L) and severe hypotension (52/37 mmHg). Biochemical data were available through July 2025, and clinical follow-up continued until June 2026. Adrenal insufficiency was not initially considered. When hypotension was first documented in November 2023 the patient was still receiving tislelizumab, and the accompanying anorexia and fatigue were attributed to treatment; by the November 2024 admission tislelizumab had been stopped eleven months earlier, and the symptoms were attributed to the underlying malignancy. Paired morning ACTH and cortisol were therefore not obtained until December 2024. Compared with previous reports, this case contributes a fully documented recognition pathway together with a more severe presentation and longer follow-up, rather than a new endocrine phenotype. This pattern is consistent with a recent systematic review of ICI-associated adrenal insufficiency, which found that early symptoms are often vague, easily misattributed to cancer-related or treatment-related causes, and associated with delayed hormonal testing and diagnosis (5). Therefore, persistent or clustered nonspecific symptoms in ICI-exposed patients should prompt early morning ACTH and cortisol testing, even when symptoms are initially mild or attributed to malignancy-related causes.

**Table 3:** Comparison of 12 previously reported patients with tislelizumab-associated adrenal insufficiency or pituitary–adrenal axis dysfunction and the present case.

Case	Patient, malignancy, PD-L1	Tislelizumab-containing regimen	Onset and presentation	Key endocrine evidence	Diagnosis and diagnostic certainty	Adrenal crisis reported (Y/N)	Glucocorticoid replacement and endocrine outcome
Baek, 2022 Case 1 (25)	58/M; stage IV HER2-negative gastric adenocarcinoma with portal-vein tumor thrombosis; PD-L1 NR	Capecitabine + oxaliplatin + tislelizumab 200 mg/m^2^ every 3 weeks (dose reproduced as reported)	After cycle 6: severe fatigue; Sodium 109 mmol/L	Cortisol <0.5 µg/dL; ACTH 20.23 pg/mL; cosyntropin cortisol 2.27/3.56 µg/dL at 30/60 min; severe primary hypothyroidism	Adrenal insufficiency; impaired stimulation with inappropriately normal ACTH suggests a central pattern, but localization was not fully established; pituitary MRI normal	N	Hydrocortisone 10 mg on awakening/5 mg early evening plus levothyroxine 0.15 mg daily; endocrine follow-up 6 weeks, axis recovery not assessed; tislelizumab continued, no interruption reported
Baek, 2022 Case 2 (25)	59/M; stage IV HER2-negative gastric adenocarcinoma with para-aortic nodal and liver metastases; PD-L1 NR	6 cycles capecitabine + oxaliplatin + tislelizumab, then 7 cycles capecitabine + tislelizumab	After cycle 13: severe weakness, mild dizziness; Sodium 123 mmol/L	Cortisol 0.79 µg/dL; ACTH 51.90 pg/mL; TSH 8.28 mIU/L; stimulation testing and pituitary MRI NR	Adrenal insufficiency with possible central pattern; anatomic localization not established	N	Hydrocortisone 10 mg twice daily plus levothyroxine 0.05 mg daily; at 6 weeks random cortisol 9.15 µg/dL on replacement; endocrine follow-up 6 weeks, axis recovery not assessed; treatment restarted after stabilization
Zhang, 2023 (18)	72/M; T1N0M0 bladder urothelial carcinoma plus T2N0M0 prostate adenocarcinoma; PD-L1 NR	Tislelizumab 200 mg q3w × 5; abiraterone/goserelin; intravesical gemcitabine × 7	Symptoms began about 1 month after the last dose: nausea/vomiting, anorexia, fatigue, functional decline, 19-kg weight loss; Sodium 130 mmol/L	Cortisol <0.50 µg/dL; ACTH <3 pg/mL; urinary free cortisol 11.8 µg/24 h; hypogonadism; primary hypothyroidism	Tislelizumab-induced hypophysitis with SAI; MRI showed pituitary fullness/heterogeneous signal and PET/CT showed stalk thickening with increased uptake	N	IV hydrocortisone followed by oral hydrocortisone 20 mg twice daily; biochemical improvement was reported at 2 months (ACTH 8.2 pg/mL; cortisol 7.8 µg/dL), but endogenous HPA-axis recovery could not be confirmed during ongoing replacement; further ICI use NR.
Wei, 2024 (17)	58/M; recurrent low-grade non-invasive papillary urothelial carcinoma; PD-L1 NR	Tislelizumab 200 mg every 3 weeks; approximately 8 cycles	Recurrent syncope for >3 months with fever, confusion, fatigue, anorexia, nausea/vomiting; episodic SBP 50–60 mmHg; Sodium 142 mmol/L at admission	Cortisol 0.16/0.20/0.15 µg/dL and ACTH 4.47/2.37/2.16 pg/mL (08:00/16:00/24:00); other pituitary axes largely preserved	Central AI/isolated ACTH deficiency presenting as grade 3–4 adrenal crisis; adrenal CT and pituitary MRI normal	Y	IV hydrocortisone sodium succinate 150 mg twice daily with fluid resuscitation, then prednisone 10 mg AM/5 mg PM; no further syncope over 3 months, but no repeat ACTH or cortisol reported, so recovery not assessed; tislelizumab stopped and switched to an ADC
Wang, 2024 Case 1 (24)	51/M; stage IVA right lung squamous cell carcinoma; heavy smoker; PD-L1 NR	Nab-paclitaxel 200 mg days 1/8 + carboplatin 550 mg + tislelizumab 200 mg every 21 days	At cycle 4; asymptomatic laboratory finding	Synchronous low morning cortisol and ACTH; increased TSH; additional pituitary-axis abnormalities; stimulation testing not performed	SAI; pituitary MRI normal	N	Prednisone acetate 5 mg twice daily plus levothyroxine 50 µg daily; thyroid function normalized after 2 months, while morning cortisol remained subnormal during >8 months of replacement; endogenous recovery was not established; ICI deferred and not restarted
Wang, 2024 Case 2 (24)	74/M; stage IVA left lung squamous cell carcinoma with rib metastasis; smoker; PD-L1 NR	One initial combination cycle; later third-line tislelizumab 200 mg every 21 days x 9, with a final dose plus denosumab	At cycle 9; asymptomatic laboratory finding	Synchronous low morning cortisol and ACTH; electrolytes and thyroid tests normal; stimulation testing and pituitary MRI not performed	AI with a central biochemical pattern; hypophysitis not radiologically assessed	N	Prednisone acetate 5 mg AM/2.5 mg PM; morning cortisol remained subnormal during >7 months of replacement; endogenous recovery was not established; ICI deferred and not restarted
Wang, 2024 Case 3 (24)	56/M; stage IIIA right lung squamous cell carcinoma; diabetes and smoking history; PD-L1 NR	Nab-paclitaxel 200 mg days 1/8 + carboplatin 500 mg + tislelizumab 200 mg every 21 days for 4 cycles, then tislelizumab maintenance	At cycle 8: fatigue, vomiting, 2.5-kg weight loss in 1 month, and rash	Low morning cortisol and ACTH; thyroid tests/electrolytes normal; stimulation testing and pituitary MRI not performed	Central-pattern AI; hypophysitis unconfirmed	N	Glucocorticoid replacement; cortisol normalized and ACTH increased after 2 months, suggesting apparent biochemical recovery. After a single-cycle tislelizumab rechallenge, cortisol decreased to 43 nmol/L, replacement was restarted, and tislelizumab had not been resumed at the last follow-up.
Deng, 2024 (19)	67/M; invasive high-grade bladder urothelial carcinoma; PD-L1 NR	Gemcitabine 1.8 g days 1/8 + cisplatin 120 mg day 2 + tislelizumab 200 mg day 4 every 21 days	After cycle 2: psoriasis-like rash, fluid/sodium retention, electrolyte disturbance, hypoalbuminemia, fever, nausea/vomiting, and fatigue	Cortisol <0.1 then 0.143 µg/dL; ACTH <1 pg/mL; adrenal CT normal	Central-pattern AI; pituitary MRI normal; hypophysitis unconfirmed	N	Oral methylprednisolone 8 mg daily with symptomatic improvement; at about 5 weeks, cortisol remained <0.1 µg/dL and ACTH <1 pg/mL during ongoing replacement, so endogenous recovery could not be assessed; tislelizumab was suspended, with no subsequent use reported.
Zhang, 2025 Case 2 (20)	47/M; stage IV B3 thymoma with pleural involvement; PD-L1 not measured	Cycles 1–3 doxorubicin/cyclophosphamide/cisplatin; cycles 4–6 same chemotherapy + tislelizumab 200 mg every 3 weeks; cycles 7–15 tislelizumab	Cortisol fell 40% from baseline after the first ICI cycle; grade 2 AI recorded across cycles 5-7; symptoms NR	Exact cortisol, ACTH, electrolytes, dynamic testing, and pituitary imaging NR	Grade 2 immune-related AI; subtype NR	N	Systemic corticosteroid replacement from May to September 2023, well controlled; drug, dose, ACTH and follow-up cortisol NR, tislelizumab maintenance continued
Hu, 2025 (21)	69/F; recurrent metastatic endometrial carcinosarcoma; PD-L1 NR	Tislelizumab 200 mg + anlotinib x 5 cycles	Somnolence 25 days and delirium 40 days after the last dose; poor appetite/vomiting; Sodium 129 mmol/L; eosinophilia	Cortisol <1.00 µg/dL and ACTH <5 pg/mL at 08:00/16:00; other pituitary hormones initially normal	Central AI/possible isolated ACTH deficiency; normal pituitary/stalk on cranial MRI; clinically attributed to ICI-related hypophysitis.	Y	Hydrocortisone 100 mg every 12 h, tapered to oral maintenance; a later procedure-associated adrenal crisis responded to 100 mg IV hydrocortisone. Levothyroxine was added for subsequent hypothyroidism; follow-up was approximately 6 weeks, and HPA-axis recovery was not assessed; subsequent ICI use NR.
Liu, 2025 (22)	47/F; heavily pretreated recurrent high-grade serous ovarian carcinoma; PD-L1 CPS <1; prior toripalimab exposure	Tislelizumab + nab-paclitaxel + carboplatin x 6 from Nov 2021, then tislelizumab + olaparib maintenance from May 2022	Feb 2023 (about 15 months after starting tislelizumab): anorexia, fatigue, poor sleep	Cortisol 1.71 µg/dL; ACTH 2.59 pmol/L (inappropriately normal); urine cortisol 1 µg/24 h; imaging/dynamic tests NR	AI, localization uncertain; confounded by prior PD-1 therapy and multisystem irAEs	N	Hydrocortisone (dose NR) with symptomatic improvement, plus levothyroxine for earlier hypothyroidism; repeat ACTH and cortisol NR; all antitumor treatment was stopped in April 2023 when grade 4 pneumonitis developed.
Sheng, 2026 Index case (23)	56/M; esophageal squamous cell carcinoma; PD-L1 NR	Nab-paclitaxel 400 mg + carboplatin 500 mg + tislelizumab 200 mg: 3 neoadjuvant and 4 adjuvant cycles; then tislelizumab maintenance × 12 (Apr 2023–Mar 2024).	During/after maintenance: fatigue, anorexia, polyuria/polydipsia, dizziness; Na 121.7 mmol/L; exact onset interval NR	Cortisol <0.4/0.4/<0.4 µg/dL; ACTH 1.92/<1/1.07 pg/mL; central hypothyroidism, secondary hypogonadism/GH deficiency, autoimmune diabetes	ICI-related panhypopituitarism with SAI plus autoimmune diabetes; pituitary MRI slightly flattened; adrenal CT normal	N	Prednisone acetate 5 mg AM/2.5 mg PM, levothyroxine 150 µg daily, and basal-bolus insulin; symptoms and serum sodium normalized at 2 months, but HPA-axis recovery was not assessed; the 12-cycle tislelizumab maintenance course had been completed before presentation, and subsequent ICI use was NR.
Present case	66/M; resected stage IIB (pT1cN1M0) lung squamous cell carcinoma; 40 pack-years; PD-L1 not tested	Carboplatin 0.5 g + paclitaxel liposome 210 mg + tislelizumab 200 mg x 4 cycles, then tislelizumab 200 mg every 4 weeks x 13 maintenance cycles; last dose 11 Dec 2023	Anorexia from Nov 2023, followed by fatigue, headache, nausea/vomiting and 19-kg weight loss; later BP 75/56 then 52/37 mmHg; Sodium 108.9 mmol/L; glucose 3.38 mmol/L	No pretreatment ACTH/cortisol baseline. On 11 Dec 2024: morning cortisol <1.00 µg/dL and ACTH <5.00 pg/mL; thyroid function normal; no cosyntropin test	Tislelizumab-associated SAI (Naranjo score 6, probable); non-contrast head CT showed no metastasis or macroadenoma; pituitary MRI not performed, so hypophysitis was not confirmed	Y	IV hydrocortisone 50 mg AM/25 mg PM with fluid resuscitation, followed by oral prednisone maintenance; symptoms, blood pressure, and electrolyte abnormalities improved; no recurrent adrenal crisis was reported through June 2026, although endogenous HPA-axis recovery could not be reliably assessed

ACTH, adrenocorticotropic hormone; ADC, antibody–drug conjugate; AI, adrenal insufficiency; CPS, combined positive score; HPA, hypothalamic-pituitary-adrenal; ICI, immune checkpoint inhibitor; NR, not reported; PD-L1, programmed death-ligand 1; SAI, secondary adrenal insufficiency; SBP, systolic blood pressure. All entries were verified against the full text of the source publications. “Adrenal crisis reported” indicates whether the source publication described hypotension or shock, syncope, or acute deterioration requiring parenteral glucocorticoid rescue; Y indicates that such an episode was described; N indicates that no such episode was described and does not exclude its occurrence. NR, not reported.

In this patient, septic, cardiogenic, and hypovolemic shock were considered; however, these alternative causes were not supported by the overall clinical course, inflammatory markers, cardiac evaluation, imaging findings, and the rapid clinical improvement after hydrocortisone replacement with supportive treatment. Once ICI-associated SAI is diagnosed, patients require glucocorticoid replacement, education regarding stress-dose glucocorticoids, and structured endocrine follow-up to assess treatment adequacy and potential HPA axis recovery (9).

This case report has several limitations. Pituitary MRI and complete assessment of the remaining pituitary axes were not available; therefore, hypophysitis could not be confirmed, isolated ACTH deficiency could not be distinguished from broader pituitary dysfunction, and the exact site of HPA-axis injury remained uncertain. Cosyntropin stimulation testing was not performed, and follow-up blood sampling was not standardized relative to prednisone administration, precluding reliable assessment of endogenous HPA-axis recovery. As a single case report, causality cannot be definitively established, and the findings should be interpreted in the context of existing literature.

## Conclusions

This case highlights tislelizumab-associated secondary adrenal insufficiency as a potentially underrecognized endocrine immune-related adverse event. In patients receiving or previously exposed to tislelizumab, unexplained fatigue, gastrointestinal symptoms, hyponatremia, hypoglycemia, weight loss, or hypotension should prompt early measurement of paired morning ACTH and cortisol levels. Timely glucocorticoid replacement and structured endocrine follow-up are essential to help prevent adrenal crisis, guide long-term management, and assess potential HPA axis recovery.

## Patient perspective

The patient reported significant improvement in quality of life after glucocorticoid replacement, with resolution of fatigue, nausea, and vomiting. He was educated on the need for long-term glucocorticoid replacement, stress-dose adjustment, and ongoing endocrine follow-up.

## Data Availability

The original contributions presented in the study are included in the article. Further inquiries can be directed to the corresponding author.
